# Effect of Astragaloside IV on improving cardiac function in rats with heart failure: a preclinical systematic review and meta-analysis

**DOI:** 10.3389/fphar.2023.1226008

**Published:** 2023-10-03

**Authors:** Zhiyuan Zhang, Muxin Zhang, Yongkai Xu, Mengkai Lu, Lei Zhang, Chao Li

**Affiliations:** ^1^ Innovation Research Institute of Traditional Chinese Medicine, Shandong University of Traditional Chinese Medicine, Jinan, China; ^2^ First Clinical Medical College, Shandong University of Traditional Chinese Medicine, Jinan, China; ^3^ Department of Peripheral Vascular Medicine, Affiliated Hospital of Shandong University of Traditional Chinese Medicine, Jinan, China; ^4^ College of Traditional Chinese Medicine, Shandong University of Traditional Chinese Medicine, Jinan, China

**Keywords:** Astragaloside IV, heart failure, cardiac function, preclinical studies, meta-analysis

## Abstract

**Background:** Astragaloside IV (ASIV) is the primary pharmacologically active compound found in *Astragalus propinquus* Schischkin, which has potential protective effects on cardiac function. However, there are almost no systematic evaluations of ASIV for the treatment of heart failure (HF).

**Methods:** Preclinical studies published before 27 December 2022, were retrieved from PubMed, Web of Science, MEDLINE, SinoMed, Chinese National Knowledge Infrastructure (CNKI), VIP information database, and Wanfang Data information site. The quality of included research was evaluated using SYRCLE’s RoB tool. Review Manager 5.4.1 was used to perform meta-analyses of the cardiac function parameters and other indicators. Regression analysis was conducted to observe the dose-efficacy relationship.

**Results:** Nineteen studies involving 489 animals were included. Results indicated that compared with the control group, ASIV could enhance cardiac function indicators, including left ventricular ejection fraction (LVEF), left ventricular fractional shortening (LVFS), left ventricular pressure change rate (±dp/dt_max_), left ventricular end-diastolic pressure (LVEDP), left ventricular systolic pressure (LVSP), heart weight/body weight (HW/BW) and left ventricular weight/body weight (LVW/BW). Furthermore, the regression analysis showed that the treatment of HF with ASIV was dose-dependent.

**Conclusion:** Findings suggest that ASIV can inhibit cardiac hypertrophy by reducing cardiac preload and afterload, thereby protecting cardiac function.

## 1 Introduction

Heart failure (HF) is a complex clinical syndrome that develops due to structural or functional damage of ventricular filling or ejection of blood, and it represents an advanced manifestation of various cardiovascular diseases (Heidenreich et al., 2022). The American Heart Association predicts that by 2030, HF will probably affect more than 8 million people over 18 years old in the United States (Heidenreich et al., 2013). In China, epidemiological surveys report a 4.1% ± 0.3% in-hospital mortality rate for HF (Zhang et al., 2017). Mortality and incidence rates increase with age (Huffman et al., 2013), which contributes to a rising economic burden from HF as the population ages (Cook et al., 2014). Despite significant progress in treatments that have improved the survival of HF patients, such as angiotensin-converting enzyme inhibitors, angiotensin receptor blockers, β receptor blockers, coronary arterial blood revascularization, implantable cardioverter-defibrillators, and cardiac resynchronization therapy (Merlo et al., 2014; Vaduganathan et al., 2020), the 5-year mortality rate of HF remains high (Gerber et al., 2015).

**FIGURE 1:**
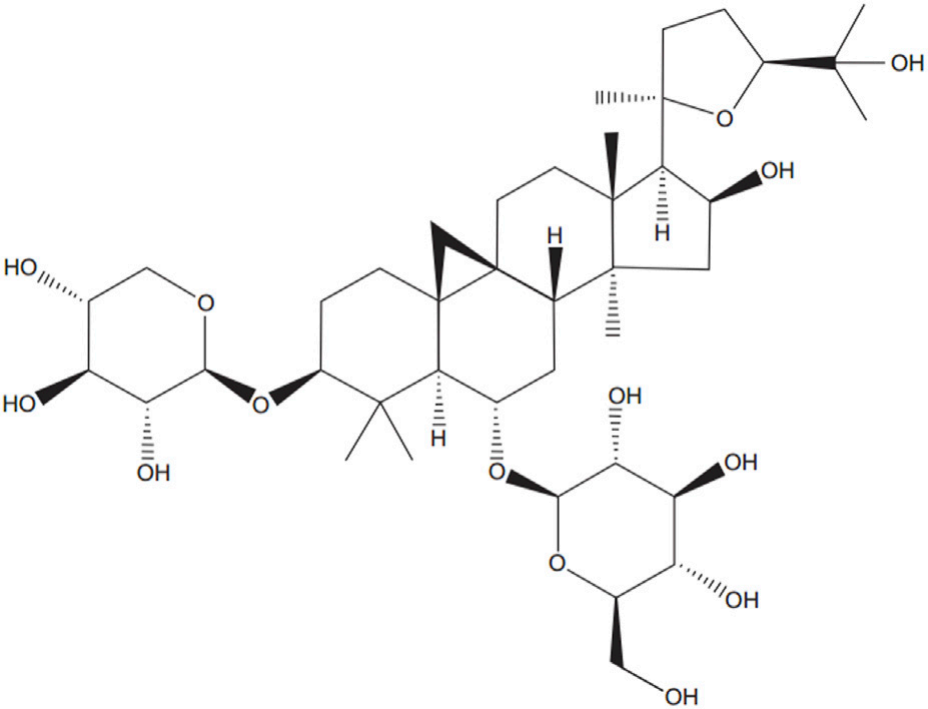
Chemical structure of Astragaloside IV.

Recent clinical studies suggest that natural medicine could significantly improve the prognosis of HF patients (Wang et al., 2018; Mao et al., 2020; Leung et al., 2021). *Astragalus propinquus* Schischkin, widely used in traditional Chinese clinical prescriptions, frequently features in prescriptions for treating HF (Guo et al., 2022). In a recent study, Huangqi injection (with active ingredients derived from *Astragalus propinquus* Schischkin) demonstrated the ability to improve cardiac function (Cao et al., 2022). Huangqi injection was also found to significantly improve various parameters of echocardiography in rats with heart failure, including LVEF and LVFS (Liu et al., 2018). Recent animal studies have shown that Astragaloside IV (ASIV) (Figure 1), the active ingredient of *Astragalus propinquus* Schischkin, can protect cardiovascular system (Dong et al., 2017; Liu et al., 2021). Accumulating evidence indicates that ASIV can promote angiogenesis (Wang et al., 2013), protect myocardial cells (Luo et al., 2019), and inhibit ventricular remodeling (Lu et al., 2017).

Preclinical systematic reviews can identify areas for testing in further animal experiments, preclude unnecessary study replication, refine animal experimentation, and lay the foundation for future clinical trials (Murphy and Murphy, 2010). Therefore, in this study, we conducted a systematic review and meta-analysis to evaluate the beneficial effects of ASIV on cardiac function in HF rat models. The results of our study could provide a reference for refining animal experimentation and designing clinical research, as well as identifying new therapeutic strategies for the treatment of HF.

## 2 Methods

This systematic review was registered (Invoice Number: CRD42023383485) in PROSPERO (https://www.crd.york.ac.uk/PROSPERO/) and has been reported in line with the Preferred Reporting Items for Systematic Reviews and Meta-Analyses (PRISMA).

### 2.1 Search strategies

We conducted a comprehensive search of studies on the effect of ASIV in animal models of HF using various electronic databases, such as PubMed, Web of Science, MEDLINE, SinoMed, Chinese National Knowledge Infrastructure (CNKI), VIP information database, and Wanfang Data information site, from their inception to December 2022.

The following keywords combined with Medical Subject Headings (MeSH) terms were used for searching: (“Astragaloside IV” or “ASIV” or “astragaloside-A”) AND (“heart failure” or “HF” or “cardiac failure” or “Heart Decompensation” or “Myocardial Failure”).

### 2.2 Inclusion/exclusion criteria

To prevent bias, prespecified inclusion criteria were as follows:

(1) rat models of HF, without limiting specific modeling method;
(2) a controlled experiment;
(3) treatment group received the ASIV intervention merely, administration of ASIV at any dose or in any form is acceptable;
(4) control group received equivalent vehicle, saline or no treatment;
(5) the outcomes measured were parameters reflecting cardiac function, such as left ventricular ejection fraction (LVEF), and/or left ventricular fractional shortening (LVFS), and/or left ventricular pressure change rate (LV ± dp/dt_max_), and/or left ventricular end-diastolic pressure (LVEDP) and/or left ventricular systolic pressure (LVSP) and/or heart weight/body weight (HW/BW) and/or left ventricular weight/body weight (LVW/BW).

Prespecified exclusion criteria met anyone of the following conditions:

(1) *in vitro* studies, case reports, and clinical trials;
(2) duplicate publications;
(3) Missing result data that can be obtained.

### 2.3 Data extraction

Two authors independently extracted data as follows: 1) the first author’s name and publication year; 2) the information of experimental animals such as number, species, sex, weight age; 3) the induction method of HF animal model; 4) the time of experimental drug intervention; 5) the information of treatment used in experimental group such as dose, method of administration, and duration of treatment; 6) the primary outcome measures. If there were multiple measurement results at different times, we recorded the last result. If the experimental animals received different doses of drug intervention, we recorded only the highest dose. The data were measured by the digital ruler software if the data was presented with graphs. For incomplete published data, we contacted the author for further information. For each comparison, we extracted the mean and standard deviation from the experimental and control groups of each study. Discrepancies were resolved after discussion between the two authors.

### 2.4 Outcome

The data for LVEF and LVFS were obtained through echocardiography measurements. The data for LVEDP, LVSP, and ±dp/dt_max_ were obtained through hemodynamic monitoring. The data for HW/BW and LVW/BW were obtained through post-mortem measurements and calculations.

### 2.5 Quality assessment

We evaluated the methodological quality of the included studies using the SYRCLE’s RoB tool (Hooijmans et al., 2014) with minor modification as follows: 1) randomization of sequence generation; 2) description of baseline characteristics; 3) allocation concealment; 4) animals randomly standardized housed; 5) feeding and intervention in blind; 6) criterion for the success of animal models; 7) random outcome assessment; 8) blinded assessment of outcomes; 9) incomplete outcome data; (10) Other sources of bias. We tried to quantify the evaluation results. Each study was given a total score of ten, with one point for each entry. Two authors independently evaluated the study quality, and disagreement was resolved through discussion or consultation.

### 2.6 Statistical analysis

We performed a meta-analysis using Review Manager 5.4.1. All the data of cardiac function were considered as continuous data, and then, we use mean deviation (MD) and random effect model (REM) to estimate the size of combined effects. Because of the heterogeneity between multiple studies must be considered, in this meta-analysis, we chose the REM to get the results. The *χ* ^*2*^ test with a significance level of = 0.1 will be used as statistical measure of heterogeneity between the different studies. Moreover, the *I* ^*2*^ statistic will be applied to quantifies inconsistency between studies, calculated as *I* ^*2*^ = (*Q* - *df*)/Q*100%, where *I* ^*2*^ statistic of 50% or more indicated a considerable heterogeneity, then additional subgroup and/or sensitivity analysis was performed. Probability values of 0.05 were considered significant. In addition, Origin 2021 was used for dosage-efficacy interval analyses, and regression analysis was used to test the reliability of the dosage-efficacy interval.

## 3 Results

### 3.1 Study inclusion

**FIGURE 2:**
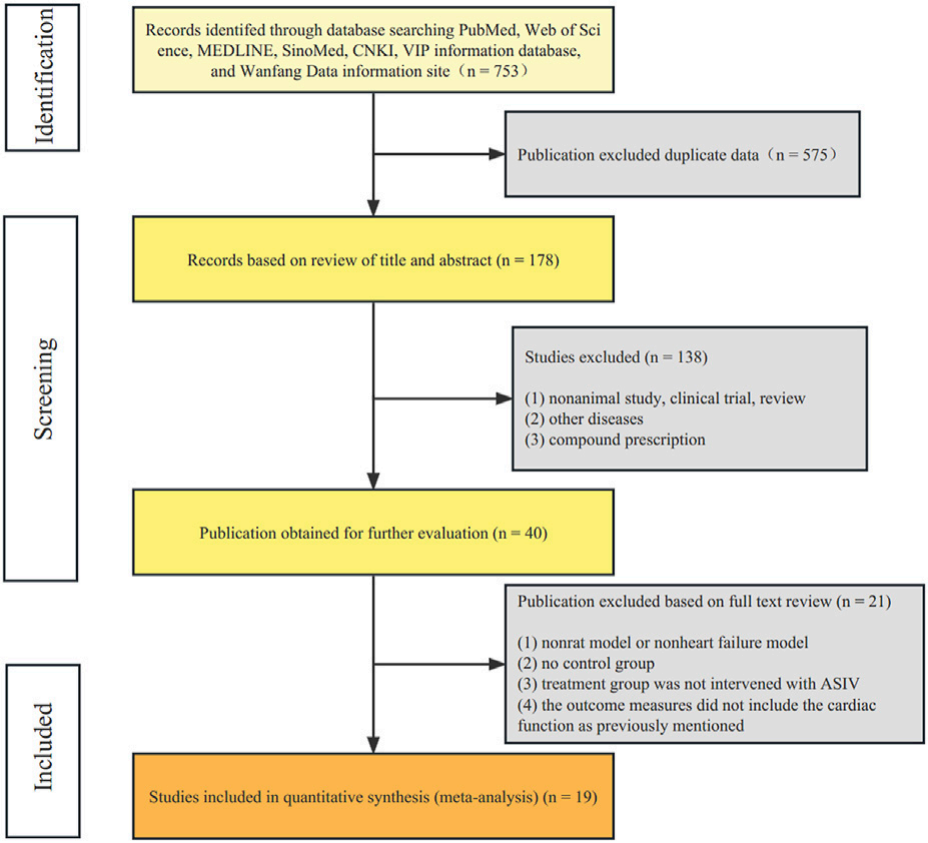
Flow diagram. The process of papers inclusion was divided into three steps: search, deduplication, and manual screening. Only literature that met the inclusion criteria were included.

According to our retrieval strategy, we identified 753 potentially relevant studies. There were 178 records after deleting duplicates. After reading the titles and abstracts, 138 studies were excluded because of non-animal study, clinical trial, review, other diseases, or compound prescription. The full text of the remaining 40 articles was read, and finally 21 articles were excluded for the following reasons: 1) non-rat model or non-heart failure model; 2) no control group; 3) treatment group was not intervened with ASIV; 4) the outcome measures did not include the cardiac function as previously mentioned. Ultimately, 19 randomized controlled animal experiments (Zhao et al., 2009; Cui et al., 2013; Zhang et al., 2015; Cheng et al., 2016; Jiang et al., 2016; Cheng, 2017; Ji et al., 2018; Lv, 2018; Tang et al., 2018; Zhao et al., 2018; Nie et al., 2019; Sui et al., 2020; Wang et al., 2020; Huang et al., 2021; Shi et al., 2021; Song et al., 2021; Zhang et al., 2021; Cui et al., 2022; Wang et al., 2022) were identified (Figure 2).

### 3.2 Study characteristics

A total of 489 animals were included in 19 studies. All studies were published in peer-reviewed journals. All studies were published between 2009 and 2022, including eight English studies (Zhao et al., 2009; Zhang et al., 2015; Cheng et al., 2016; Ji et al., 2018; Tang et al., 2018; Nie et al., 2019; Sui et al., 2020; Shi et al., 2021). Male Sprague Dawley rats were used in fifteen studies (Cui et al., 2013; Zhang et al., 2015; Cheng et al., 2016; Jiang et al., 2016; Cheng, 2017; Lv, 2018; Tang et al., 2018; Zhao et al., 2018; Sui et al., 2020; Wang et al., 2020; Huang et al., 2021; Shi et al., 2021; Song et al., 2021; Zhang et al., 2021; Wang et al., 2022), and male Wistar rats were used in two studies (Ji et al., 2018; Cui et al., 2022). In the remaining studies, one study (Zhao et al., 2009) used Wistar rats but did not report gender, and one study (Cui et al., 2022) did not report the type and gender of rats used. All studies reported animal weights. As for the rat model of HF, nine studies (Zhao et al., 2009; Cui et al., 2013; Cheng et al., 2016; Cheng, 2017; Ji et al., 2018; Sui et al., 2020; Wang et al., 2020; Shi et al., 2021; Wang et al., 2022) used the method of coronary artery ligation, six studies (Jiang et al., 2016; Lv, 2018; Tang et al., 2018; Zhao et al., 2018; Nie et al., 2019; Song et al., 2021) used abdominal aortic coarctation (AAC), and other methods included injection of isoproterenol (Zhang et al., 2015), injection of miRNA-1 lentivirus (Huang et al., 2021), injection of doxorubicin (Zhang et al., 2021), and high salt feeding (Cui et al., 2022). Thirteen studies (Zhao et al., 2009; Cui et al., 2013; Jiang et al., 2016; Cheng, 2017; Ji et al., 2018; Tang et al., 2018; Zhao et al., 2018; Nie et al., 2019; Sui et al., 2020; Shi et al., 2021; Song et al., 2021; Zhang et al., 2021; Wang et al., 2022) mentioned that the specific time of starting intervention was after modeling, and two studies (Zhang et al., 2015; Huang et al., 2021) mentioned that intervention was before modeling, the other four studies (Cheng et al., 2016; Lv, 2018; Wang et al., 2020; Cui et al., 2022) did not indicate the specific time of intervention. The administration methods include intravenous injection and gastric perfusion, and only one study (Zhao et al., 2009) used intravenous injection. The dose was not exactly the same, including the following: 80 mg/kg/d in four studies (Zhang et al., 2015; Nie et al., 2019; Huang et al., 2021; Shi et al., 2021); 70 mg/kg/d in two studies (Cheng, 2017; Lv, 2018); 60 mg/kg/d in four studies (Jiang et al., 2016; Tang et al., 2018; Zhao et al., 2018; Zhang et al., 2021); 50 mg/kg/d in two studies (Cheng et al., 2016; Song et al., 2021); 40 mg/kg/d in one study (Cui et al., 2022); 30 mg/kg/d in one study (Wang et al., 2022); 20 mg/kg/d in one study (Ji et al., 2018); 10 mg/kg/d in one study (Cui et al., 2013); 2 mg/kg/d in one study (Wang et al., 2020); 1 mg/kg/d in two studies (Zhao et al., 2009; Sui et al., 2020). The durations of administration time are diverse, including 56 days in eight studies (Cheng, 2017; Lv, 2018; Tang et al., 2018; Nie et al., 2019; Wang et al., 2020; Song et al., 2021; Zhang et al., 2021; Cui et al., 2022); 28 days in six studies (Zhang et al., 2015; Jiang et al., 2016; Ji et al., 2018; Zhao et al., 2018; Shi et al., 2021; Wang et al., 2022); 14 days in two studies (Zhao et al., 2009; Cheng et al., 2016); the remaining three studies different from each other.

Eleven studies (Cheng et al., 2016; Tang et al., 2018; Zhao et al., 2018; Nie et al., 2019; Sui et al., 2020; Wang et al., 2020; Huang et al., 2021; Song et al., 2021; Zhang et al., 2021; Cui et al., 2022; Wang et al., 2022) reported LVEF; eight studies (Zhao et al., 2009; Zhang et al., 2015; Cheng et al., 2016; Tang et al., 2018; Nie et al., 2019; Sui et al., 2020; Wang et al., 2020; Wang et al., 2022) reported LVFS; nine studies (Zhao et al., 2009; Cui et al., 2013; Zhang et al., 2015; Jiang et al., 2016; Cheng, 2017; Tang et al., 2018; Zhao et al., 2018; Shi et al., 2021; Song et al., 2021) reported LV + dp/dt; ten studies (Zhao et al., 2009; Cui et al., 2013; Zhang et al., 2015; Jiang et al., 2016; Cheng, 2017; Ji et al., 2018; Tang et al., 2018; Zhao et al., 2018; Shi et al., 2021; Song et al., 2021) reported LV-dp/dt; eight studies (Zhao et al., 2009; Cui et al., 2013; Zhang et al., 2015; Jiang et al., 2016; Cheng, 2017; Lv, 2018; Zhao et al., 2018; Shi et al., 2021) reported LVSP; twelve studies (Zhao et al., 2009; Cui et al., 2013; Zhang et al., 2015; Jiang et al., 2016; Cheng, 2017; Ji et al., 2018; Lv, 2018; Tang et al., 2018; Zhao et al., 2018; Wang et al., 2020; Shi et al., 2021; Song et al., 2021) reported LVEDP; five studies (Cheng et al., 2016; Jiang et al., 2016; Zhao et al., 2018; Nie et al., 2019; Wang et al., 2022) reported HW/BW; four studies (Jiang et al., 2016; Zhao et al., 2018; Song et al., 2021; Wang et al., 2022) reported LVW/BW. The main characteristics of the 19 studies are summarized in Table 1.

**TABLE 1:** Characteristics of the included studies.

Study (years)	Species (Sex; *n* = experimental/control group)	Weight	Random method	Model (method)	Time drug given	Treatment group	Control group	Outcome index	Intergroup differences
Zhao et al. (2009)	Wistar rats (unknown; *n* = 10/10)	228.3 2813.0 g	Not described	By coronary ligation	The treatment started 3 weeks after coronary ligation	By intravenous injection; AS-IV (1 mg/kg; qd) for 14 d	By intravenous injection; NS (equal doses; qd) for 14 d	1.LVFS	1.*p* <0.01
								2.LV .Ldp/dt	2.*p* <0.01
								3.LVEDd	3.*p* <0.01
								4.LVESD	4.*p* <0.01
								5.LVSP	5.*p* <0.01
								6.LVEDP	6.*p* <0.01
Cui et al. (2013)	SD rats (male; *n* = 20/20)	200∼250 g	The random number table	By coronary ligation	The treatment started 2 days after coronary ligation	By intragastric administration; AS-IV (10 mg/kg; qd) for 40 d	By intragastric administration; NS (equal doses; qd) for 40 d	1.LV .Ldp/dt	1.*p* <0.01
								2.LVSP	2.*p* <0.01
								3.LVEDP	3.*p* <0.01
Cheng (2017)	SD rats (male; *n* = 10/10)	220 2010 g	Not mentioned	By coronary ligation	The treatment started 7 weeks after coronary ligation	By intragastric administration; AS-IV (70 mg/kg; qd) for 56 d	By intragastric administration; NS (1mL; qd) for 56 d	1.LV .Ldp/dt	1.*p* <0.05
								2.LVSP	2.*p* <0.05
								3.LVEDP	3.*p* <0.05
Wang et al. (2020)	SD rats (male; *n* = 20/20)	230 3010 g	The random number table	By coronary ligation	Not mentioned	By intragastric administration; AS-IV (2 mg/kg; qd) for 56 d	By intragastric administration; distilled water (equal doses; qd) for 56 d	1.LVEF	1.*p* <0.05
								2.LVFS	2.*p* <0.05
								3.LVEDP	3.*p* <0.05
Sui et al. (2020)	SD rats (male; *n* = 15/15)	200∼220 g	Not described	By coronary ligation	The treatment started 1 day after coronary ligation	By intragastric administration; AS-IV (1 mg/kg; qd) for 42 d	By intragastric administration; NS (equal doses; qd) for 42 d	1.LVEF	1.*p* <0.01
								2.LVFS	2.*p* <0.01
								3.LVEDd	3.*p* <0.01
								4.LVESD	4.*p* <0.01
Wang et al. (2022)	SD rats (male; *n* = 10/10)	200∼220 g	Not described	By coronary ligation	The treatment started 1 hour after coronary ligation	By intragastric administration; AS-IV (30 mg/kg; qd) for 28 d	By intragastric administration; NS (equal doses; qd) for 28 d	1.LVEF	1.*p* <0.05
								2.LVFS	2.*p* <0.05
								3.LVEDd	3.*p* <0.05
								4.LVESD	4.*p* <0.05
								5.HW/BW	5.*p* <0.05
								6.LVW/BW	6.*p* <0.05
Cheng et al. (2016)	SD rats (male; *n* = 15/15)	250 5020 g	Not described	By coronary ligation	Not mentioned	By intragastric administration; AS-IV (50 mg/kg; qd) for 14 d	By intragastric administration; NS (equal doses; qd) for 14 d	1.LVEF	1.*p* <0.01
								2.LVFS	2.*p* <0.01
								3.LVEDd	3.*p* <0.01
								4.LVESD	4.*p* <0.01
								5.HW/BW	5.*p* <0.01
Shi et al. (2021)	SD rats (male; *n* = 10/10)	200 0020 g	Not described	By coronary ligation	The treatment started 2 weeks after coronary ligation	By intragastric administration; AS-IV (80 mg/kg; qd) for 28 d	By intragastric administration; deionized water (equal doses; qd) for 28 d	1.LV .Ldp/dt	1.*p* <0.01
								2.LVSP	2.*p* <0.01
								3.LVEDP	3.*p* <0.01
Ji et al. (2018)	Wistar rats (male; *n* = 8/8)	200∼250 g	Not described	By coronary ligation	The treatment started 5 weeks after coronary ligation	By intragastric administration; AS-IV (20 mg/kg; qd) for 28 d	By intragastric administration; NS (equal doses; qd) for 28 d	1.LV-dp/dt	1.*p* <0.05
								2.LVEDP	2.*p* <0.05
Tang et al. (2018)	SD rats (male; *n* = 20/20)	210 1010 g	The random number table	By abdominal aortic constriction	The treatment started 6 weeks after abdominal aortic constriction	By intragastric administration; AS-IV (60 mg/kg; qd) for 56 d	By intragastric administration; 1% sodium carboxymethyl cellulose (1mL; qd) for 56 d	1.LVEF	1.*p* <0.01
								2.LVFS	2.*p* <0.01
								3.LV .Ldp/dt	3.*p* <0.01
								4.LVEDd	4.*p* <0.05
								5.LVESD	5.*p* <0.05
								6.LVEDP	6.*p* <0.01
Zhang et al. (2015)	SD rats (male; *n* = 10/10)	180∼200 g	Not described	By injecting Iso	The treatment started 2 weeks before Iso injection	By intragastric administration; AS-IV (80 mg/kg; qd) for 28 d	By intragastric administration; 1% sodium carboxymethyl cellulose (equal doses; qd) for 28 d	1.LVFS	1.*p* <0.01
								2.LV .Ldp/dt	2.*p* <0.01
								3.LVSP	3.*p* <0.01
								4.LVEDP	4.*p* <0.01
Huang et al. (2021)	SD rats (male; *n* = 8/8)	240 4020 g	Not described	By injecting miRNA-1 lentivirus in left ventricular wall	The treatment started 1 week before miRNA-1 lentivirus injection	By intragastric administration; AS-IV (80 mg/kg; qd) for 21 d	By intragastric administration; NC(equal doses; qd) for 21 d	1.LVEF	1.*p* <0.01
								2.LVFS	2.*p* <0.01
Zhang et al. (2021)	SD rats (male; *n* = 10/10)	200 0030 g	Not described	By injecting Dox	The treatment started 2 weeks after Dox injection	By intragastric administration; AS-IV (60 mg/kg; qd) for 56 d	By intragastric administration; distilled water (equal doses; qd) for 56 d	1.LVEF	1.*p* <0.01
								2.LVEDd	2.*p* <0.01
								3.LVESD	3.*p* <0.01
Zhao et al. (2018)	SD rats (male; *n* = 21/21)	261.73 6113.28 g	The random number table	By abdominal aortic constriction	The treatment started 8 weeks after abdominal aortic constriction	By intragastric administration; AS-IV (60 mg/kg; qd) for 28 d	By intragastric administration; distilled water (20 mg/kg; qd) for 28 d	1.LVEF	1.*p* <0.05
								2.LV .Ldp/dt	2.*p* <0.05
								3.LVEDd	3.*p* <0.05
								4.LVESD	4.*p* <0.05
								5.LVSP	5.*p* <0.05
								6.LVEDP	6.*p* <0.05
								7.HW/BW	7.*p* <0.05
								8.LVW/BW	8.*p* <0.05
Jiang et al. (2016)	SD rats (male; *n* = 12/12)	280∼300 g	Not mentioned	By abdominal aortic constriction	The treatment started 8 weeks after abdominal aortic constriction	By intragastric administration; AS-IV (60 mg/kg; qd) for 28 d	By intragastric administration; NC(2 mL; qd) for 28 d	1.LV .Ldp/dt	1.*p* <0.01
								2.LVSP	2.*p* <0.01
								3.LVEDP	3.*p* <0.01
								4.HW/BW	4.*p* <0.01
								5.LVW/BW	5.*p* <0.01
Cui et al. (2022)	Salt-sensitive rats (male; *n* = 5/5)	200 0020 g	Not described	By high salt feed	Not mentioned	By intragastric administration; AS-IV (40 mg/kg; qd) for 56 d	By intragastric administration; distilled water (equal doses; qd) for 56 d	1.LVEF	1.*p* <0.05
								2.LVEDd	2.*p* >0.05
Lv (2018)	SD rats (male; *n* = 20/20)	218.86 1810.78 g	Not described	By abdominal aortic constriction	Not mentioned	By intragastric administration; AS-IV (70 mg/kg; qd) for 56 d	By intragastric administration; NC(2mL; qd) for 56 d	1.LVEDd	1.*p* <0.01
								2.LVESD	2.*p* <0.01
								3.LVSP	3.*p* <0.01
								4.LVEDP	4.*p* <0.01
								5.LVW/BW	5.*p* <0.01
Song et al. (2021)	SD rats (male; *n* = 10/11)	260 6020 g	Not described	By abdominal aortic constriction	The treatment started 12 hours after abdominal aortic constriction	By intragastric administration; AS-IV (50 mg/kg; qd) for 56 d	By intragastric administration; 1% sodium carboxymethyl cellulose (equal doses; qd) for 56 d	1.LVEF	1.*p* <0.05
								2.LV .Ldp/dt	2.*p* <0.05
								3.LVEDd	3.*p* <0.05
								4.LVESD	4.*p* <0.05
								5.LVEDP	5.*p* <0.05
Nie et al. (2019)	Unknown (unknown; *n* = 10/10)	240 ± 10 g	The random number table	By abdominal aortic constriction	The treatment started 8 weeks after abdominal aortic constriction	By intragastric administration; AS-IV (80 mg/kg; qd) for 56 d	By intragastric administration; 1% DMSO (1mL; qd) for 56 d	1.LVEF	1.*p* <0.05
								2.LVFS	2.*p* <0.05
								3.LVEDd	3.*p* <0.05
								4.LVESD	4.*p* <0.05
								5.HW/BW	5.*p* <0.05

### 3.3 Study quality

The quality of the study included 19 studies with quality scores ranging from 2 to 6, with an average of 3.8. Five studies (Cui et al., 2013; Tang et al., 2018; Zhao et al., 2018; Nie et al., 2019; Wang et al., 2020) adopted random number table method to the groups, and the other two studies (Jiang et al., 2016; Cheng, 2017) did not mention randomization. No research has described the method and process of randomization in detail. One study (Tang et al., 2018) detailed the baseline of animal characteristics. No study described allocation concealment and random placement of animals. Seven studies (Cui et al., 2013; Cheng et al., 2016; Jiang et al., 2016; Cheng, 2017; Ji et al., 2018; Lv, 2018; Huang et al., 2021) did not mention specific environments regarding animal feeding. Nonetheless, it is a pity that none of these studies reported blinding of feeding and intervention. Twelve studies (Zhao et al., 2009; Jiang et al., 2016; Cheng, 2017; Ji et al., 2018; Lv, 2018; Tang et al., 2018; Zhao et al., 2018; Nie et al., 2019; Huang et al., 2021; Song et al., 2021; Zhang et al., 2021; Cui et al., 2022) used the results of hemodynamics and/or ultrasonic cardiogram as the standard for evaluating HF models; six studies (Cui et al., 2013; Cheng et al., 2016; Sui et al., 2020; Wang et al., 2020; Shi et al., 2021; Wang et al., 2022) used results of ECG; one study (Zhang et al., 2015) did not report evaluation methods. One study (Nie et al., 2019) mentioned blind evaluation of results, but none of the studies assessed the outcomes randomly. One study (Sui et al., 2020) did not include all animals in the final data. As anesthetic, six studies (Cui et al., 2013; Cheng et al., 2016; Cheng, 2017; Sui et al., 2020; Song et al., 2021; Wang et al., 2022) used pentobarbital sodium, three studies (Tang et al., 2018; Nie et al., 2019; Shi et al., 2021) used isoflurane, five studies (Jiang et al., 2016; Ji et al., 2018; Zhao et al., 2018; Wang et al., 2020; Huang et al., 2021) used chloral hydrate, one study (Zhao et al., 2009) used ether, one study (Zhang et al., 2015) used urethane, and three studies (Lv, 2018; Zhang et al., 2021; Cui et al., 2022) did not report the use of drugs. No study reports conflicts of interest. The methodological quality of each study is summarized in Table 2.

**TABLE 2:** The quality of included studies.

Study	1	2	3	4	5	6	7	8	9	10	Total
Zhao et al. (2009)	√			√		√			√	√	5
Cui et al. (2013)	√								√	√	3
Cheng (2017)						√			√	√	3
Wang et al. (2020)	√			√					√		3
Sui et al. (2020)	√			√					√	√	4
Wang et al. (2022)	√			√					√	√	4
Cheng et al. (2016)	√								√	√	3
Shi et al. (2021)	√			√					√	√	4
Ji et al. (2018)	√					√			√		3
Tang et al. (2018)	√	√		√		√			√	√	6
Zhang et al. (2015)	√			√					√		3
Huang et al. (2021)	√					√			√		3
Zhang et al. (2021)	√			√		√			√		4
Zhao et al. (2018)	√			√		√			√		4
Jiang et al. (2016)						√			√		2
Cui et al. (2022)	√			√		√			√		4
Lv (2018)	√					√			√		3
Song et al. (2021)	√			√		√			√	√	5
Nie et al. (2019)	√			√		√		√	√	√	6

Notes: Studies fulfilling the criteria of: 1. Randomization of sequence generation; 2. Description of baseline characteristics; 3. Allocation concealment; 4. Animals randomly standardized housed; 5. Feeding and intervention in blind; 6. Criterion for the success of animal models; 7. Random outcome assessment; 8. Blinded assessment of outcomes; 9. Incomplete outcome data; 10. Other sources of bias.

### 3.4 Outcomes

#### 3.4.1 LVEF (%)

**FIGURE 3:**
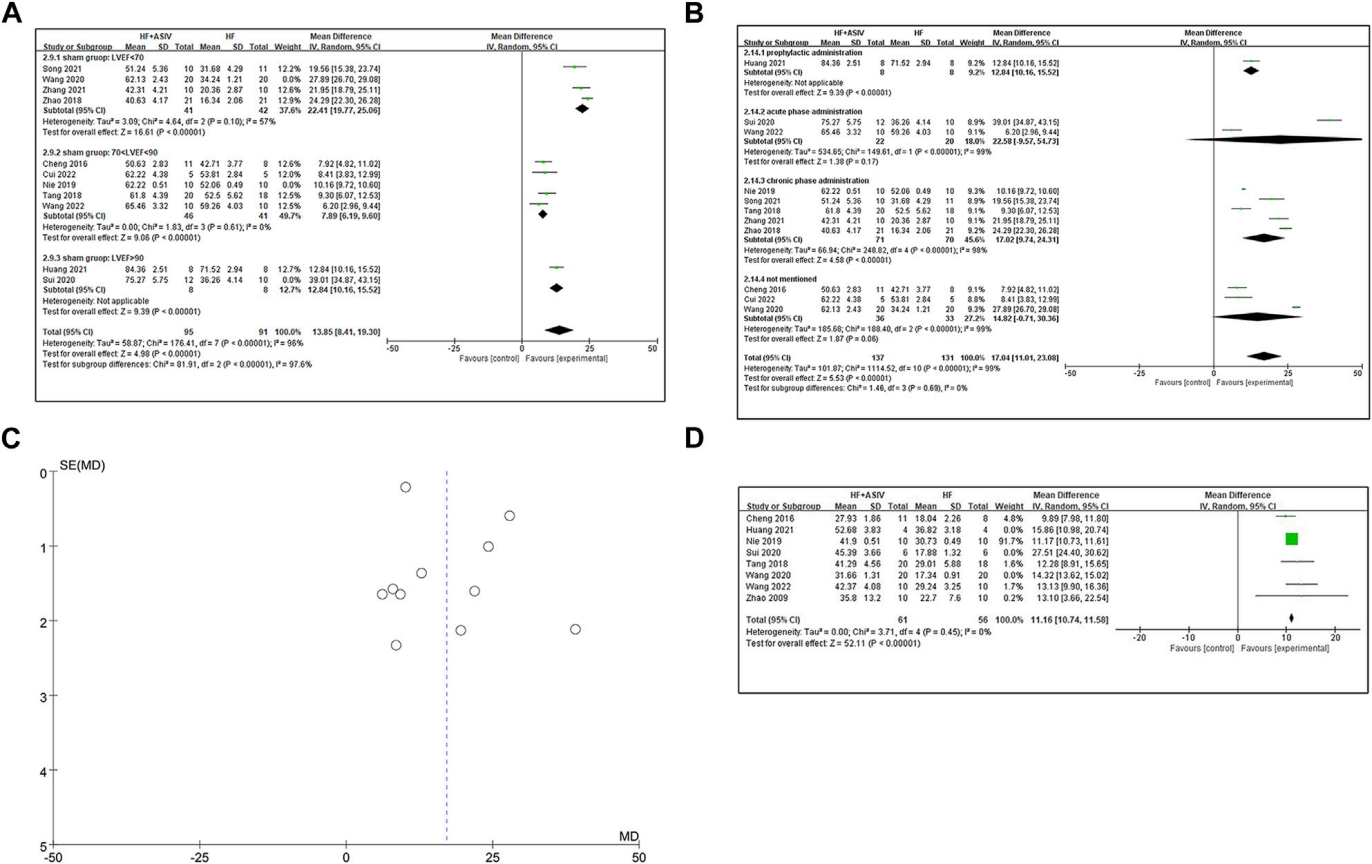
**(A)** The forest plot: subgroup analysis of ASIV in sham group (LVEF <70%), sham group (70% < LVEF <90%) and sham group (LVEF >90%) for improving LVEF compared with the control group. **(B)** The forest plot: subgroup analysis of ASIV in prophylactic administration group, acute phase administration group, chronic phase administration group and not mentioned group for improving LVEF compared with the control group. **(C)** Funnel plot indicating a predominantly symmetrical distribution of the 11 included studies assessing the outcome of LVEF. **(D)** The forest plot: effects of ASIV for increasing LVFS compared with the control group.

Eleven studies (Cheng et al., 2016; Tang et al., 2018; Zhao et al., 2018; Nie et al., 2019; Sui et al., 2020; Wang et al., 2020; Huang et al., 2021; Song et al., 2021; Zhang et al., 2021; Cui et al., 2022; Wang et al., 2022) reported LVEF, and the results of meta-analysis showed that ASIV had a significant effect on improving LVEF compared with the control group (*n* = 268, MD 17.04, 95% CI: 11.01∼23.08, *p* < 0.01; *heterogeneity Chi* ^*2*^ = 1114.52, *p* < 0.01, *I* ^*2*^ = 99%). Due to significant statistical heterogeneity, we considered using subgroup analysis to explore the sources of heterogeneity. We noticed that the LVEF results of the control group were significantly different in the included studies. Among the sham group results of these studies, there are five studies (Cheng et al., 2016; Tang et al., 2018; Nie et al., 2019; Cui et al., 2022; Wang et al., 2022) for 70% < LVEF <90%, four studies (Zhao et al., 2018; Wang et al., 2020; Song et al., 2021; Zhang et al., 2021) for LVEF <70%, and two studies (Sui et al., 2020; Huang et al., 2021) for LVEF >90%. Subgroup analysis showed that, in sham group (LVEF <70%) (Zhao et al., 2018; Wang et al., 2020; Song et al., 2021; Zhang et al., 2021) (*n* = 123, MD 23.78, 95% CI: 20.28∼27.28, *p* < 0.01; *heterogeneity Chi* ^*2*^ = 27.92, *p* < 0.01, *I* ^*2*^ = 89%), sham group (70% < LVEF <90%) (Cheng et al., 2016; Tang et al., 2018; Nie et al., 2019; Cui et al., 2022; Wang et al., 2022) (*n* = 107, MD 8.84, 95% CI: 7.20∼10.48, *p* < 0.01; *heterogeneity Chi* ^*2*^ = 8.17, *p* = 0.09, *I* ^*2*^ = 51%) and sham group (LVEF >90%) (Sui et al., 2020; Huang et al., 2021) (*n* = 38, MD 25.88, 95% CI: 0.23∼51.52, *p* = 0.05; *heterogeneity Chi* ^*2*^ = 108.07, *p* < 0.01, *I* ^*2*^ = 99%), ASIV improved LVEF more than control group. It suggests that the baseline characteristics of animals may be the potential cause of heterogeneity. Next, considering that the difference in total drug dose is huge (mean = 1935.82, standard deviation = 1395.01), we removed three studies (Nie et al., 2019; Sui et al., 2020; Wang et al., 2020) (dose >4000 mg/kg or <500 mg/kg). Subgroup analysis showed that, in sham group (LVEF <70%) (Zhao et al., 2018; Song et al., 2021; Zhang et al., 2021) (n = 83, MD 22.41, 95% CI: 19.77∼25.06, *p* < 0.01; *heterogeneity Chi* ^*2*^ = 4.64, *p* = 0.10, *I* ^*2*^ = 57%) and sham group (70 < LVEF <90) (Cheng et al., 2016; Tang et al., 2018; Cui et al., 2022; Wang et al., 2022) (n = 87, MD 7.89, 95% CI: 6.19∼9.60, *p* < 0.01; *heterogeneity Chi* ^*2*^ = 1.83, *p* = 0.61, *I* ^*2*^ = 0%), ASIV improved LVEF more than control group (Figure 3A). An absence of heterogeneity test in sham group (LVEF >90%) because only a single study was included. Therefore, the drug dose may also be the potential cause of heterogeneity. Additionally, due to the different initiation times of drug administration, we conducted subgroup analyses based on another classification. The analyses were performed separately for “prophylactic administration,” “acute phase administration,” “chronic phase administration,” and “not mentioned.” The results revealed a high level of heterogeneity (Figure 3B). The differences in drug administration timing may not explain the source of heterogeneity. The symmetrical shape of the funnel plot suggests a relatively balanced inclusion of studies, implying minimal publication bias (Figure 3C).

#### 3.4.2 LVFS (%)

Eight studies (Zhao et al., 2009; Tang et al., 2018; Nie et al., 2019; Sui et al., 2020; Wang et al., 2020; Huang et al., 2021; Shi et al., 2021; Wang et al., 2022) reported LVFS, and the results of meta-analysis showed that ASIV had a significant effect on improving LVEF compared with the control group (*n* = 177, MD 14.55, 95% CI: 12.05∼17.06, *p* < 0.01; *heterogeneity Chi* ^*2*^ = 157.54, *p* < 0.01, *I* ^*2*^ = 96%). Because of the high heterogeneity, we conducted sensitivity analysis to find the source of heterogeneity. Considering the previously mentioned baseline, after excluding three studies (Sui et al., 2020; Wang et al., 2020; Huang et al., 2021) with LVEF >90% or LVEF <70% in the sham group, the results of the remaining five studies (Zhao et al., 2009; Cheng et al., 2016; Tang et al., 2018; Nie et al., 2019; Wang et al., 2022) showed that ASIV could significantly improve LVFS (*n* = 117, MD 11.16, 95% CI: 10.74∼11.58, *p* < 0.01; *heterogeneity Chi* ^*2*^ = 3.71, *p* = 0.45, *I* ^*2*^ = 0%) (Figure 3D).

#### 3.4.3 LV ± dp/dt_max_ (10^3^ mmHg/s)

**FIGURE 4:**
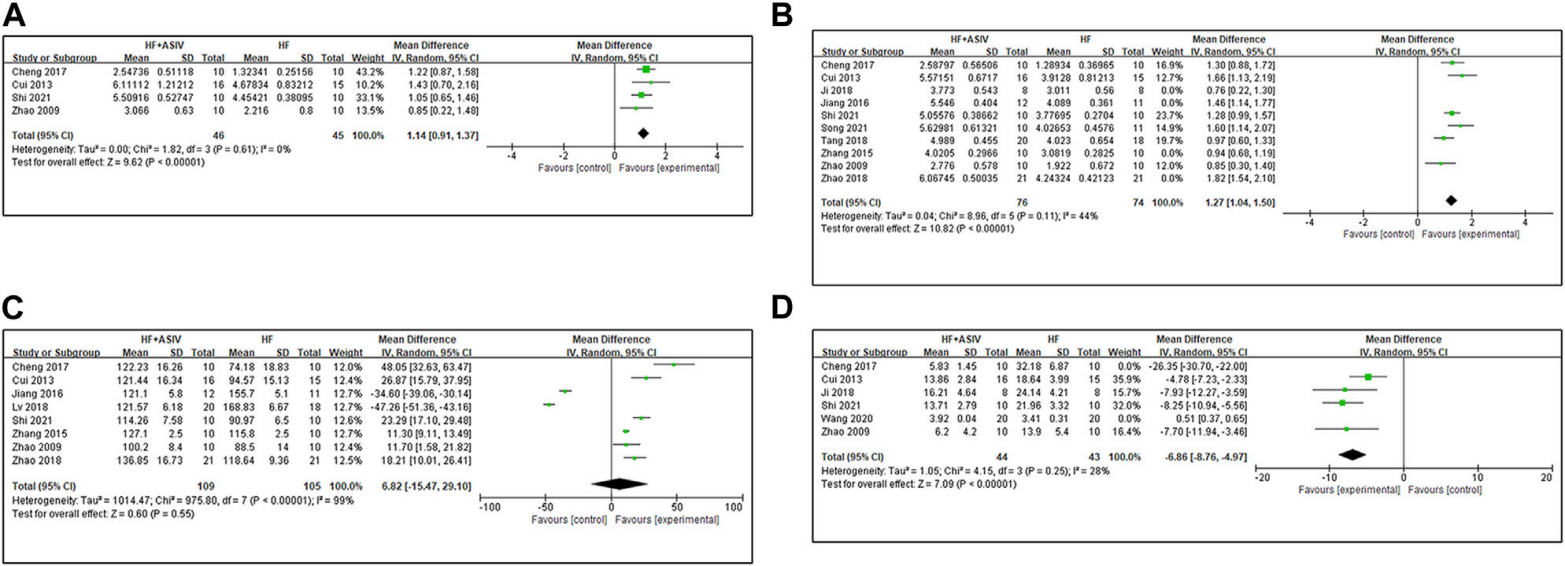
**(A)** The forest plot: effects of ASIV for increasing LV + dp/dt compared with the control group. **(B)** The forest plot: effects of ASIV for increasing LV - dp/dt compared with the control group. **(C)** The forest plot: effects of ASIV for increasing LVSP compared with the control group. **(D)** The forest plot: effects of ASIV for increasing LVEDP compared with the control group.

Nine studies (Zhao et al., 2009; Cui et al., 2013; Zhang et al., 2015; Jiang et al., 2016; Cheng, 2017; Tang et al., 2018; Zhao et al., 2018; Shi et al., 2021; Song et al., 2021) reported LV + dp/dt_max_, and the results of meta-analysis showed that ASIV had a significant effect on improving LV + dp/dt_max_ compared with the control group (*n* = 235, MD 1.19, 95% CI: 0.89∼1.49, *p* < 0.01; *heterogeneity Chi* ^*2*^ = 58.01, *p* < 0.01, *I* ^*2*^ = 86%). Furthermore, we established a subgroup analysis based on induction method of animal model due to the remarkable heterogeneity among the various studies. Four studies (Zhao et al., 2009; Cui et al., 2013; Cheng, 2017; Shi et al., 2021) used coronary artery ligation, four studies (Zhao et al., 2018; Shi et al., 2021; Song et al., 2021; Wang et al., 2022) used AAC, and one study (Zhang et al., 2015) used injection of Iso. Meta-analysis of four studies (Zhao et al., 2009; Cui et al., 2013; Cheng, 2017; Shi et al., 2021) using coronary ligation showed that ASIV could significantly improve LV + dp/dt_max_ (*n* = 91, MD 1.14, 95% CI: 0.91∼1.37, *p* < 0.01; *heterogeneity Chi* ^*2*^ = 1.82, *p* = 0.61, *I* ^*2*^ = 0%) (Figure 4A). In the four studies using abdominal aortic constriction, the heterogeneity improvement was poor (*n* = 124, MD 1.23, 95% CI: 0.62∼1.84, *p* < 0.01; *heterogeneity Chi* ^*2*^ = 46.41, *p* < 0.01, *I* ^*2*^ = 94%). Ten studies (Zhao et al., 2009; Cui et al., 2013; Zhang et al., 2015; Jiang et al., 2016; Cheng, 2017; Ji et al., 2018; Tang et al., 2018; Zhao et al., 2018; Shi et al., 2021; Song et al., 2021) reported LV - dp/dt_max_, and the results of meta-analysis showed that ASIV had a significant effect on improving LV - dp/dt_max_ compared with the control group (*n* = 251, MD 1.28, 95% CI: 1.04∼1.51, *p* < 0.01; *heterogeneity Chi* ^*2*^ = 35.25, *p* < 0.01, *I* ^*2*^ = 74%). Sensitivity analysis is used to explore the source of heterogeneity. As an anesthetic, after excluding one study on the use of urethane and three studies on the use of chloral hydrate, the meta-analysis of six studies (Zhao et al., 2009; Cui et al., 2013; Cheng, 2017; Tang et al., 2018; Shi et al., 2021; Song et al., 2021) on the use of other anesthetics (ether, isoflurane or pentobarbital sodium) showed that ASIV could significantly improve LV - dp/dt_max_ (*n* = 150, MD 1.27, 95% CI: 1.04∼1.50, *p* < 0.01; *heterogeneity Chi* ^*2*^ = 8.96, *p* < 0.11, *I* ^*2*^ = 44%) (Figure 4B). It suggests that anesthetics may be the potential cause of heterogeneity.

#### 3.4.4 LVSP (mmHg)

Eight studies (Zhao et al., 2009; Cui et al., 2013; Zhang et al., 2015; Jiang et al., 2016; Cheng, 2017; Lv, 2018; Zhao et al., 2018; Shi et al., 2021) reported LVSP, and the results of meta-analysis showed that ASIV could not be considered to increase LVSP (*n* = 214, MD 6.82, 95% CI: −15.47∼29.10, *p* = 0.55; *heterogeneity Chi* ^*2*^ = 975.80, *p* < 0.01, *I* ^*2*^ = 99%) (Figure 4C). High heterogeneity may be due to different methods of modeling or different anesthetics. Because of high heterogeneity and subgroup analysis and sensitivity analysis cannot reasonably explain the source of heterogeneity, we consider qualitative analysis. Two studies (Jiang et al., 2016; Lv, 2018) reported that ASIV decreased LVSP compared with the control group (*p* < 0.01). LVSP decreases in HF (Walley, 2016). We noticed that in these two studies, LVSP in the HF model group was higher than that in the sham group. The author did not explain or analyze this in the results. It may be the compensatory increase caused by AAC (Katz et al., 2019). Hence, we consider that it is inappropriate to combine the results of these two studies with other studies After excluding these two studies, the other six studies (Zhao et al., 2009; Cui et al., 2013; Zhang et al., 2015; Cheng, 2017; Zhao et al., 2018; Shi et al., 2021) reported that ASIV had a positive effect on reducing LVSP compared with the control group (*p* < 0.01 or *p* < 0.05).

#### 3.4.5 LVEDP (mmHg)

Twelve studies (Zhao et al., 2009; Cui et al., 2013; Zhang et al., 2015; Jiang et al., 2016; Cheng, 2017; Ji et al., 2018; Lv, 2018; Tang et al., 2018; Zhao et al., 2018; Wang et al., 2020; Shi et al., 2021; Song et al., 2021) reported LVEDP, and the results of meta-analysis showed that ASIV had a significant effect on improving LVEDP compared with the control group (*n* = 329, MD -11.59, 95% CI: −17.35∼−5.84, *p* < 0.01; *heterogeneity Chi* ^*2*^ = 5572.36, *p* < 0.01, *I* ^*2*^ = 100%). In order to explore the source of heterogeneity, we conducted subgroup analysis. Six studies (Zhao et al., 2009; Cui et al., 2013; Cheng, 2017; Ji et al., 2018; Wang et al., 2020; Shi et al., 2021) of coronary artery ligation modeling were included in the meta-analysis, and the results showed that ASIV had a significant effect on improving LVEDP compared with the control group (*n* = 147, MD -8.92, 95% CI: −15.46∼-2.38, *p* = 0.008; *heterogeneity Chi* ^*2*^ = 232.55, *p* < 0.01, *I* ^*2*^ = 98%). Then, we conducted sensitivity analysis to further explore the source of heterogeneity. LVEDP >15 is a common standard to judge HF (Bøkenes et al., 2008). Therefore, we excluded one study (Wang et al., 2020) with LVEDP much lower than 15 in the control group. After continuing to exclude a study (Cheng, 2017) with a large total dosage (3920 mg/kg), the results of four studies (Zhao et al., 2009; Cui et al., 2013; Ji et al., 2018; Shi et al., 2021) showed that ASIV had a significant effect on improving LVEDP compared with the control group (*n* = 87, MD -6.86, 95% CI: −8.76∼-4.97, *p* < 0.01; *heterogeneity Chi* ^*2*^ = 4.15, *p* = 0.25, *I* ^*2*^ = 28%) (Figure 4D).

#### 3.4.6 HW/BW (mg/g)

**FIGURE 5:**
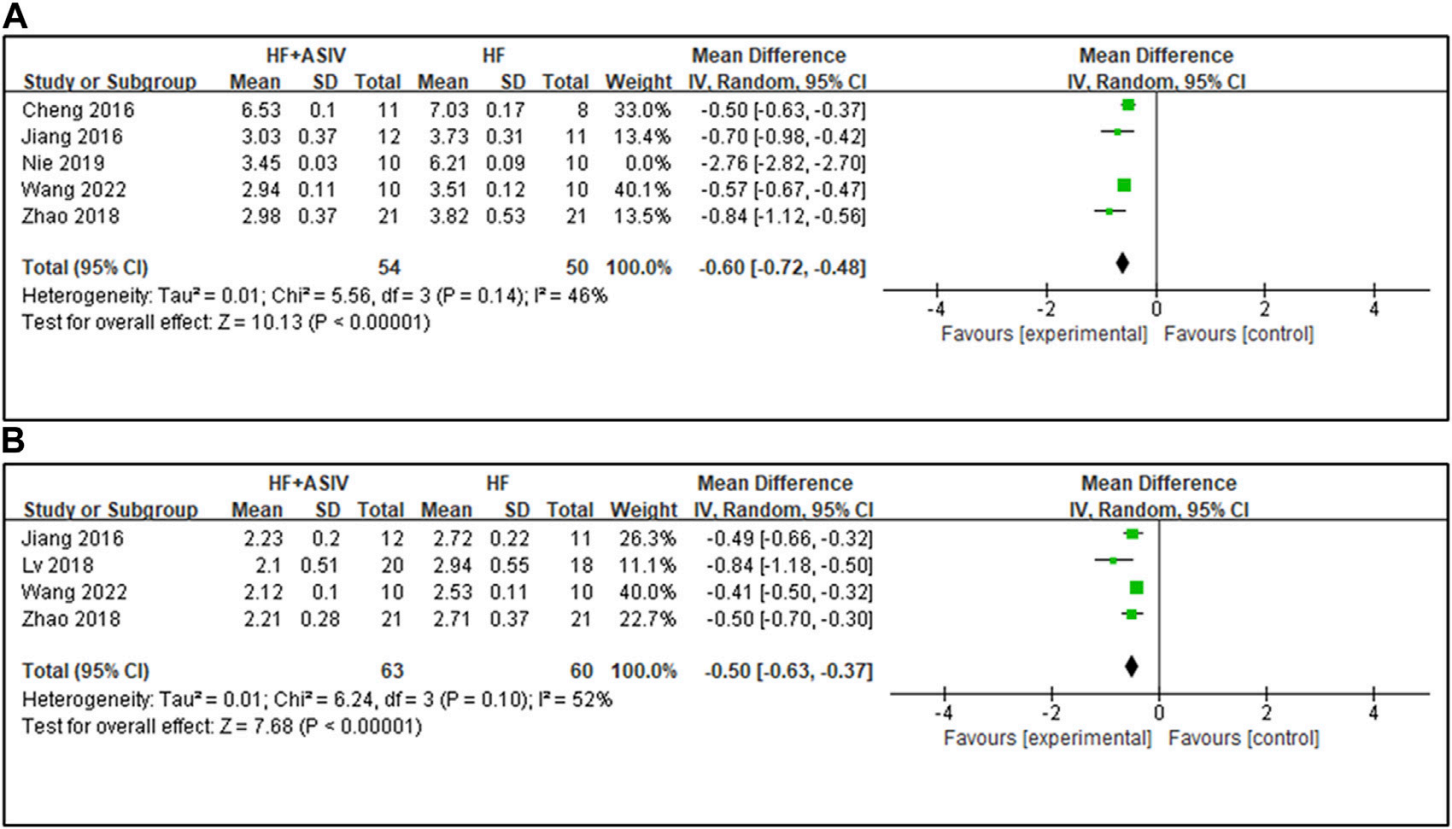
**(A)** The forest plot: effects of ASIV for reducing HW/BW compared with the control group. **(B)** The forest plot: effects of ASIV for reducing LVW/BW compared with the control group.

Five studies (Cheng et al., 2016; Jiang et al., 2016; Zhao et al., 2018; Nie et al., 2019; Wang et al., 2022) reported HW/BW, and the results of meta-analysis showed that the effect of ASIV on reducing HW/BW was not statistically significant compared with the control group (*n* = 124, MD -1.08, 95% CI: −2.32∼0.17, *p* = 0.9; *heterogeneity Chi* ^*2*^ = 2050.18, *p* < 0.01, *I* ^*2*^ = 100%). After excluding a study (Nie et al., 2019) with the largest total dose (4480 mg/kg), the results of meta-analysis of four studies (Cheng et al., 2016; Jiang et al., 2016; Zhao et al., 2018; Wang et al., 2022) showed that ASIV could significantly reduce HW/BW compared with the control group (*n* = 104, MD -0.60, 95% CI: −0.72∼−0.48, *p* < 0.01; *heterogeneity Chi* ^*2*^ = 5.56, *p* = 0.14, *I* ^*2*^ = 46%) (Figure 5A).

#### 3.4.7 LVW/BW (mg/g)

Four studies (Jiang et al., 2016; Lv, 2018; Zhao et al., 2018; Wang et al., 2022) reported LVW/BW, and the results showed that ASIV had a significant effect on improving LVW/BW compared with the control group (*n* = 123, MD -0.50, 95% CI: −0.63∼−0.37, *p* < 0.01; *heterogeneity Chi* ^*2*^ = 6.24, *p* = 0.10, *I* ^*2*^ = 52%) (Figure 5B). The reason for the high heterogeneity may be the differences in the total dose.

#### 3.4.8 Dosage-efficacy analyses

**FIGURE 6:**
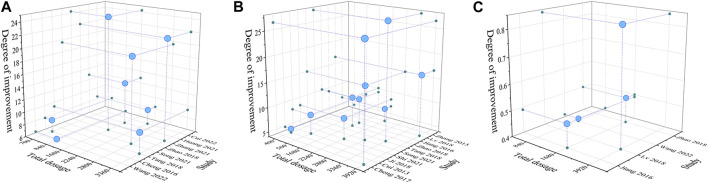
Three-dimensional images based on dosage - efficacy interval analyses [**(A)**: LVEF; **(B)** LVEDP; **(C)** LVW/BW]. The effects of ASIV on improving LVEF was not significant with the increase of dosage. The effects of ASIV on improving LVEDP and LVW/BW were enhanced with the increase of dosage.

We explored whether the total dose of ASIV would affect the improvement of cardiac function. For this reason, we selected three main indexes (LVEF, LVEDP and LVW/BW) to evaluate cardiac function and analyzed the dosage-efficacy relationship. First, we excluded the study with extremely low dose (<5 mg/kg/d). For the index of LVEDP, when the total dose of ASIV ranged from 400 mg/kg to 3920 mg/kg, the dosage-efficacy relationship shows a significant positive correlation (*Significance F* < 0.01, *p* < 0.01). However, it should be noted that for the index of LVEF, the dosage-efficacy relationship did not show a positive correlation at the dose of 700 mg/kg - 3360 mg/kg (*Significance F* > 0.05, *p* > 0.05). For the index of LVW/BW (mg/g), when the total dose of ASIV ranged from 840 mg/kg to 3920 mg/kg, the dosage-efficacy relationship shows a significant positive correlation (*Significance F* < 0.01, *p* < 0.01) (Figure 6). These results may be affected by the mode of model establishment, the drug intervention starting time, the duration of intervention and other factors. Therefore, we consider carefully that in the range of ASIV dosage from 10 mg/kg/d to 80 mg/kg/d, the effect of treating HF may be dose-dependent and/or time-dependent, but this relationship might be nonlinear.

## 4 Discussion

### 4.1 Summary of evidence

Our meta-analysis comprised 19 studies, encompassing a total of 489 animals. Our meta-analysis demonstrates that ASIV exerts cardioprotective effects in HF, as evidenced by increased LVEF, LVFS, and LV ± dp/dt_max_, as well as decreased LVSP, LVEDP, HW/BW and LVW/BW. ASIV has been shown to enhance cardiac function post myocardial infarction by inhibiting myocardial fibrosis (Zhang et al., 2022) and promoting angiogenesis (Cheng et al., 2019). Our findings also support this conclusion as evidenced by the changes in the HW/BW and LVW/BW. The dosage-efficacy relationship of ASIV is positively correlated in a range of 10–80 mg/kg/d, indicating that higher doses and longer intervention times may be more effective in treating HF with ASIV, but this relationship may not increase linearly.

### 4.2 Highlights and limitations

This meta-analysis and systematic review evaluated the latest research on the therapeutic effects of ASIV in ameliorating heart function decline caused by HF. In the past 3 years from 2020 to 2022, eight related animal experimental studies have been published. However, there have been no recent studies reviewing and discussing animal experiments, hence our work is timely and necessary. Our study focused on targeted analyses of multiple measurements to assess the positive effects of ASIV on reducing cardiac preload and afterload while inhibiting cardiac hypertrophy under conditions of HF. The dosage-efficacy interval analyses provide valuable information for future animal experiments to determine appropriate treatment times and doses. Additionally, this study contributes to reducing duplicate animal studies, improving animal research design, and provides reference evidence for converting preclinical experimental results into clinical use.

Some limitations of the study are listed as follows. The methodological quality of the included studies is generally poor. All studies lacked descriptions of allocation concealment and random placement of animals, and there were no reports of blinding with regard to feeding or intervention. Poor methodological quality is an inherent limitation that can impact accuracy (Landis et al., 2012). Furthermore, eight studies employed chloral hydrate as an anesthetic agent. Intraperitoneal administration of chloral hydrate in rats can induce non-mechanical intestinal obstruction, peritonitis, gastric ulcers, and intraperitoneal hemorrhage, which raises ethical concerns in animal research (Silverman and Muir, 1993; Baxter et al., 2009; Percie du Sert et al., 2020). Furthermore, chloral hydrate may elicit intricate effects on the cardiovascular system, thereby compromising the reliability of the results (Laurent et al., 2006; Han et al., 2011; Grissinger, 2019). Therefore, due to the imperfections in some experimental designs, we should treat the present positive results with caution. Given that ASIV’s effect on treating HF may be multi-targeted, additional research is necessary to analyze potential mechanisms of action. Moreover, because of the small sample size, the dosage-efficacy relationship of ASIV in treating HF requires further investigation with larger sample sizes and higher-quality evidence.

### 4.3 Implications

Numerous studies have demonstrated the crucial role of high-quality animal experiments as a reference point for drugs in preclinical research prior to clinical trials. However, given the vast differences between animal models and clinical practice, meticulous attention must be paid to the experimental design in preclinical research. This systematic review highlights key considerations for researchers, including the necessity of providing detailed descriptions of baseline characteristics before and after establishing animal models, as well as the use of standardized assessments, such as the SYRCLE Risk of Bias tool and the ten-item scale, to promote methodological quality. In particular, randomization and blinding techniques should be fully employed throughout the experimental process, including during model induction and outcome assessment. In the majority of relevant *in vivo* investigations, SD rats or Wistar rats are commonly employed as animal models. However, the utilization of genetically modified mice holds paramount significance in elucidating the underlying mechanisms, thereby warranting the recommendation for a more diversified selection of genetically edited mice to explore potential mechanisms. Exploring various administration methods assumes critical importance in attaining a comprehensive understanding of drug delivery efficacy and variations, consequently enriching our overall comprehension of experimental outcomes. In light of this, we recommend including research on different administration routes to address this knowledge gap. Meanwhile, taking into account the ethics of animal experiments and the impact of anesthesia on cardiovascular indicators, we recommend the use of isoflurane or pentobarbital sodium as anesthetic agents. HF typically presents in elderly patients with underlying conditions such as hypertension. Therefore, the use of relevant animal models can enhance the meaningfulness of the results. In the treatment of HF, long-term interventions and therapies are of paramount importance (Arrigo et al., 2020). Therefore, it is equally crucial to enhance our understanding of the enduring impact of ASIV on overall prognosis by increasing relevant research, thus further investigating the clinical prospects of ASIV’s application in HF management. We stress the importance of conducting studies with a wider dose range, including grouping doses, to determine the optimal dosing regimen. Such studies are essential to improving the clinical relevance and translatability of experimental results (Singh et al., 2022).

## 5 Conclusion

ASIV, a promising natural compound, has garnered significant attention due to its anti-inflammatory, antioxidant stress, neuroprotective, and other beneficial effects (Liang et al., 2023). It has been extensively investigated for its potential therapeutic applications in cardiovascular and cerebrovascular diseases, hepatitis, cancer, and other conditions (Chen et al., 2021; Li et al., 2022). Part of the pharmacological effects of ASIV can be attributed to its hydrolyzed active metabolite, Cycloastragenol (Yu et al., 2018). Regarding pharmacokinetics, ASIV exhibits relatively low bioavailability and absorption rates in the gastrointestinal tract of rats, with an absolute bioavailability of 2.2% (Gu et al., 2004). The elimination half-life of AS-IV in rats ranges from 34.0 to 131.6 min (Zhang et al., 2006). Following intravenous administration, ASIV is rapidly absorbed and widely distributed in various tissues. The kidneys and liver show the highest concentrations of ASIV, followed by the lungs, heart, and spleen. However, ASIV has limited distribution in the brain, likely due to its poor ability to cross the blood-brain barrier (Chang et al., 2012). It is important to note that there is limited research on the drug metabolism and safety of ASIV, and the quality of existing studies is not optimal. This poses a challenge for further exploration of the clinical therapeutic effects of ASIV. Most studies have utilized relatively low dosages and short administration durations, which may not be sufficient to observe acute and chronic toxicity. Therefore, more comprehensive investigations are needed to fully understand the potential benefits and safety profile of ASIV.

Additionally, the lack of high-quality meta-analyses and systematic reviews contributes to a limited understanding of the preclinical research efficacy of ASIV. Our study presents initial preclinical evidence supporting ASIV as a promising drug candidate for HF therapy. ASIV shows potential to safeguard cardiac function by decreasing cardiac preload and afterload, as well as inhibiting myocardial hypertrophy. Notably, our dose-effect analysis indicates that ASIV’s therapeutic effects range from 10 mg/kg to 80 mg/kg daily dosage, with a possible non-linear positive relationship between the dose and the efficacy.

## Data Availability

The original contributions presented in the study are included in the article/Supplementary Material, further inquiries can be directed to the corresponding author.

## Abbreviations

ASIV, Astragaloside IV; HF, Heart failure; CNKI, Chinese National Knowledge Infrastructure; CI, confidence interval; LVEF, left ventricular ejection fraction; LVFS, left ventricular fractional shortening; ± dp/dtmax, left ventricular pressure change rate; LVEDP, left ventricular end-diastolic pressure; LVSP, left ventricular systolic pressure; HW/BW, heart weight/body weight; LVW/BW, left ventricular weight/body weight; PRISMA, Preferred reporting items for systematic reviews and meta-analyses; MeSH, Medical Subject Headings; MD, mean deviation; REM, and random effect model.
