# Supplementary material for: Effect of Astragaloside IV on improving cardiac function in rats with heart failure: a preclinical systematic review and meta-analysis
Source: Front Pharmacol. 2023 Oct 3;14:1226008. doi: 10.3389/fphar.2023.1226008 (PMC10579795; doi:10.3389/fphar.2023.1226008)
Supplement: Supplementary file 1 [file DataSheet1.docx]

**Table S1 Search strategy for PubMed**

#1 Astragaloside IV [MeSH Major Topic]

#2 (ASIV[Title/Abstract]) OR (astragaloside-A[Title/Abstract])

#3 #1 OR #2

#4 heart failure [MeSH Major Topic]

#5 (((HF) OR (cardiac failure)) OR (heart decompensation)) OR (myocardial failure)

#6 #4 OR #5

#7 #3 AND #6
